# A Comparison of Nurse Aides and Nurses Regarding the Work Competence of Nurse Aides in a Skill-Mixed Institution

**DOI:** 10.3390/healthcare9121725

**Published:** 2021-12-13

**Authors:** Hui-Chen Hsu, Hsiang-Wen Kung, Wen-Jen Chiang, Bih-O Lee, Ruey-Hsia Wang

**Affiliations:** 1 Nursing Department, Chiayi Branch, Taichung Veterans General Hospital, Taichung 40705, Taiwan; huichen0303@vghtc.gov.tw (H.-C.H.); cyrcw@vghtc.gov.tw (H.-W.K.); j054@vghtc.gov.tw (W.-J.C.); 2 College of Nursing, Kaohsiung Medical University, Kaohsiung 80708, Taiwan; wrhsia@kmu.edu.tw; 3 Faculty of Nursing, Airlangga Universitas, Surabaya 60115, Indonesia

**Keywords:** nurse aide, institutional healthcare, work competence, registered nurse

## Abstract

Objective: To compare the differences between the work competencies self-reported by nurse aides’ and those perceived by nurses. Method: A cross-sectional survey was employed. The settings were units implemented a skill mix model institution in Taiwan. The instruments consisted of the participants’ demographic data and a nurse aide work competence scale. Results: The results indicated that the nurse aides had room for improvement in terms of “problem solving” and “activity design”. The nurse aides and nurses differed significantly in terms of the nurse aides’ competence in “activity design”, as the nurse aides reported themselves to be more competent in “activity design” than reported by the nurses. Conclusion: Nurse aides should be incorporated into cross-disciplinary teams. Activity design should be handled by other healthcare providers such as physical therapists or senior social workers.

## 1. Introduction

In 2010, Japan was the leading country with the highest percentage of elderly people [1]. In 2018, the proportion of the elderly population in Taiwan had reached 14.05%, which signified that the country had officially became an aged society [2]. A higher aging index is concomitant with an increasing need for elderly care. Statistics show that the number of residents living in long-term care, respite care, and nursing facilities had risen from 45,298 in 2014 to 49,575 in 2018; while the number of nurse aides had risen annually from 13,343 in 2014 to 15,730 in 2018. Even so, the increase in the proportion of nursing aides is far less than that of elderly people living in institutional settings [3]. Elderly people have substantial demands for healthcare services, and nurse aides are among the main workforce supporting the provision of long-term and direct care services.

Following the emergence of an aged society in Taiwan, as well as the stipulation of the 2.0 Long-Term Care Services Act and its implementation policy, those who wish to be a certified nurse aide are required to be at least 16 years of age have completed a nurse aide training program [4]. According to the Guidelines Governing the Nurse Aide Skills Certification, the work competencies of nurse aides cover several domains such as physical care, routine care, housework handling, emergency and accident handling, family support, and professional ethics; and training courses should include holistic health care topics such as physiological support and hospice care [2,5,6]. As nurse aides have to tackle a wide range of complex tasks and provide service to specific people, it is important for nurse aides to possess such work competence.

### Background

Nurse aides, as known as nursing assistants, are commonly involved in caring for and assisting a patient in their daily activities. The identification of work competencies and the way nursing aides acquire these competencies are important for enhancing their skills and knowledge in managing care activities [7]. Research has shown that nursing aides should possess professional competencies such as providing physical care, providing routine care, handling emergency situations, guiding patients to engage in activities, providing psychological support, and demonstrating their professional ethics [8,9]. Lai [10] opined that core competencies such as service attitude, interpersonal communication, problem solving, professional knowledge, professional skills, work ethics, personal and professional growth are prerequisites for nurse aides. Studies centering on nursing aides concurred that the provision of “physical care” and “routine care” are the primary work competencies of nurse aides in institutional settings [8,11]. Another study found that nurse aides were most competent in promoting patient comfort, implementing immediate measures to address patients’ existing problems, ensuring patient safety, and providing care in patients’ daily lives; while they were incompetent in assisting in the discovery and handling of latent problems, implementing patient care plans in practice, and guiding patients to engage in activities [12]. Sprangers et al. [13] indicated that implementing communication skills training among nurse aides can prevent incidents of verbal and physical aggression and the development of depression among nursing home residents, while also reducing the workload of registered nurses. In the study by Lai [10], nurse aides perceived the need to improve themselves in terms of professional knowledge and personal and professional growth. Wang [8] pointed out that even though “competency in measuring vital signs” is regarded as a baseline standard in the Single-level Skills Certification, few studies have covered this competence. According to the literature, nursing aids’ competencies include their knowledge, skills and attitudes. However, nursing aides in nursing homes merely play the role of a nursing assistant and a social worker, which creates a significant gap between policy development and implementation [8].

In 2008, Taiwan began to implement a skill mix model on a trial basis that can be used flexibly by the nursing workforce [14]. The skill mix model is a task shifting-based approach, in which registered nurses guide nurse aides or nursing assistants to perform semi-professional and non-professional tasks in hospital wards [15], so as to allow for more services to be provided while utilizing minimal resources [16,17]. With the support of nurse aides, registered nurses are able to focus on tasks that require a high level of professional knowledge [18]. Research has shown that registered nurses highly support the idea of assigning routine, laborious, and other non-professional tasks to nurse aides, as this enhances the job morale and professional identity of registered nurses [19,20], and even indirectly reduces their turnover intentions [15]. However, Jacob et al. [17] found that, due to differences in the education level of caregivers, the utilization of skill mix in healthcare affects caregivers’ work competencies and problem-solving skills, which prolongs patients’ length of hospitalization, increases the likelihood of developing complications, and blurs the roles of healthcare professionals. For these reasons, Twigg et al. [21] recommended that healthcare institutions should clearly define the responsibilities and the scope of practice of nurse aides, and their tasks should be distinct from those of registered nurses. For example, the relevant training conducted by registered nurses for nurse aides should be delineated, and the effectiveness of nurse aides in caregiving should be monitored.

Nurse aides provide a major support for long-term care services. Therefore, nursing institutions should continue to focus on the adequacy of their workers’ knowledge and skills [22,23]. Registered nurses play the role of a supervisor and co-worker to nurse aids. Comparing the differences between the work competencies self-reported by nurse aides and those perceived by nurses is beneficial for enhancing the work competence of nurse aides, reducing the job stress of registered nurses, and enhancing the effectiveness of patient care.

## 2. Methods

### 2.1. Design

This study employed the cross-sectional survey method to examine the work competence of nurse aides, and to compare the differences between the nurse aides’ self-reported work competencies and registered nurses’ reports of nurse aides’ work competencies.

### 2.2. Settings

This study was conducted at a hospital-based nurse institution in Taiwan. The hospital has a total of 941 beds, of which of 628 beds were in wards where a skill mix model involving nurse aides and nurses was implemented. Nurse aides served at the hospital as outsourced workers. Every two years, the hospital would call for tenders and request successful tenders to sign the relevant contracts. The contractor would recruit nurse aides, to provide group-based and individual-based caregiving. On average, nurse aides had to care for 15 residents or six to eight patients. All nurse aides were jointly managed by their contractors and the supervisors of the units they served in. A performance appraisal was carried out every month, and the results were reported to the contractor and hospital administrators. The research settings were the 11 wards where the skill mix model was implemented in.

### 2.3. Participants

The inclusion criteria were nurse aides and registered nurses who had completed three months of service and were willing to participate in this study; the exclusion criterion was nurse aides and nurses who had yet to complete three months of service.

### 2.4. Data Collection

First, the researchers sought approval from the supervisor of each research setting. Afterwards, a meeting was convened with the head nurses of various ward units, during which the researchers explained the research purposes to them clearly, so as to achieve consistency in participant inclusion. From May to August 2019, the head nurses collected data by means of the structural survey questionnaire, which was completed by nurses and nurse aides who were willing to participate in this study. A total of 220 questionnaires were administered, and 216 were recovered (110 from nurses, 106 from nurse aides), indicating a recovery rate of 98.18%. There were 174 valid responses.

The sample size was calculated using G Power 3.1.9 before data collection. An effect size of 0.5, a coefficient power of 0.8, and a significance level (α) of 0.05 was used in this study. The sample size estimated by G Power was 64 per group. This requires a minimum total of 128 participants. This study finally included 72 nurse aides and 102 nurses, which met the required sample size.

### 2.5. Instruments

The instruments consisted of the demographic data of the nurses and nurse aides, as well as the Nurse Aides’ Work Competence Scale. Developed by Chen and Lin [12] The scale was employed in this study upon approval from its authors. Since the scale was developed 10 years ago, qualitative interviews were held with 10 nurse aides recruited via purposive sampling, and the results of the interviews were used to revise the original scale. The original scale had seven subscales and 36 items. Based on the interview results, this study revised the scale by adding one item (“Able to manage and allocate working time efficiently”) and making major revisions to seven items and minor revisions to 13 items.

The revised scale had 37 items across seven subscales, namely Interpersonal relationships and communication (Items 1–6), Problem solving (Items 7–12), Caregiving competence (Items 13–30), Work ethics (Items 21–25), Personal and professional growth (Items 26–29), Managerial capability (Items 30–34), and Activity design (Items 35–37). All the items were scored on a five-point Likert scale, in which a higher score indicated a higher level of work competence. Following the revision, five experts were invited to evaluate the scale’s expert validity to check whether the items of the tool are representative of the research items. This is central to establishing the overall validity of a scale. The resulting expert validity was revealed to be more than 80%, indicating the instrument’s validity was acceptable [24]. In this study, the Cronbach’s α of the subscales ranged from 0.77 to 0.94, and the Cronbach’s α of the overall scale was 0.96.

### 2.6. Data Analysis

The questionnaire data was first coded and processed by SPSS for Windows 22.0 statistical software (IBM, Armonk, NY, USA), after which descriptive statistics were compiled and independent sample *t*-tests were performed. The independent sample *t*-tests were used to examine the differences in the mean scores of the nurse aides and registered nurses in the work competence scale.

### 2.7. Ethical Considerations

First, the study protocol was reviewed and approved by the Institutional Review Board of the hospital. (IRB No: SE19050B). Taking into account the participants’ authority, the researchers respected the participants’ freedom and willingness to participate. The researchers explained the research objectives to the participants and guaranteed that their personal data would be kept confidential, and the participants were able to terminate their participation at any time without compromising their rights as workers. Data collection began after the researchers had obtained the participants’ approval and signed consent forms.

## 3. Results

### 3.1. Demographic Variables of Nurse Aides

The mean age of the nurse aides was 52.17 (11.22) years; a majority (84.72%) of them were female; 94.44% of them held Taiwanese citizenship; 51.39% of them were Buddhists; in terms of education level, most of them (51.39%) had completed their senior high school or vocational high school education; most of them (68.06%) were married; and most of them (65.28%) had a job tenure of six years or longer (See Table 1).

### 3.2. Demographic Variables of Nurses

The mean age of the nurses was 30.00 (8.41) years; a majority of them (98.04%) were female; in terms of education level, most of them (98.04%) held a college or higher-level degree; most of them (61.76%) were single; and most of them (60.78%) had a job tenure of six years or longer (See Table 2).

### 3.3. Descriptive Statistics Derived from the Work Competence Scale

The nurse aides perceived that they had a decent and medium-to-high level of work competence, as indicated by a mean score of 4.36 (0.67) points (Table 3). The highest-scoring items were “able to engage in lifelong learning and enhance skills in caregiving at any time” and “able to self-reflect and mitigate shortcomings in caregiving”; the lowest-scoring item was “able to evaluate the effectiveness of solving patient-related problems”.

The nurses reported that the nurse aides had a decent and medium-to-high level of work competence, as indicated by a mean score of 4.29 (0.59) points (Table 4). The nurses reported that the nurse aides had the least competence in the “activity design” subscale; the three items with lower scores were item 34 “able to communicate and cooperate with each department in the organization”, item 36 “able to assist in designing activities that meet the needs of patients”, and item 37 “able to assist in a simple evaluation of the effectiveness of implementing an activity”.

For the “problem solving” subscale, the nurses scored the nurse aides lowest for item 11 “able to evaluate the effectiveness of solving patient-related problems”. Two items in the “caregiving competence” subscale also received low scores from the nurses, namely item 18 “assist patients to engage in exercise or rehabilitation” and item 20 “able to provide patients with basic knowledge on healthcare and caregiving”.

The differences between the nurse aides and the nurses with regard to the nurse aides’ work competence scale scores are presented in Table 5. The results show that a significant difference (*p* < 0.05) exists in the “activity design” subscale, as the items in this subscale had low scores. The nurses reported that the nurse aides had a significantly lower level of competence in activity design compared to the nurse aides’ own reports.

## 4. Discussion

This study revealed that the only significant difference between the scores of the subscales in the nurse aides’ work competence scale was the “activity design” subscale. The nurses reported that the nurse aides had the least competence in “activity design”. In addition, the nurses also reported that the nurse aides’ competence in “problem solving” and “caregiving competence” were less than ideal.

The results of this study showed that the nurse aides reported themselves to be more adept in competencies such as personal and professional growth, work ethics, and managerial capabilities. This could be due to the increase in consumer consciousness, and nurse aides becoming the front-line workers when dealing with the complaints of patients’ families. In order to prevent the problems that arise during the process of caregiving, nurse aides have to fully complete their training programs and abide by job regulations. On the other hand, the nurse aides reported themselves to be least competent in “evaluating the effectiveness of solving patient-related problems”, “assisting patients to engage in exercise or rehabilitation”, and “assisting a simple evaluation of the effectiveness of implementing an activity”. In a similar vein, Lai [10] also found that in-home nurse aides’ perceptions regarding the overall importance of core competencies for in-home caregiving were higher than their actual core competence in in-home caregiving. Wu and Wang [20] revealed that registered nurses considered patient safety as an important issue. Nurse aides were mainly assigned “basic caregiving” tasks instead of “monitoring” tasks, which resulted in a lack of comprehensive and systematic education on caregiving, in addition to being unable to comprehend the antecedents and consequences of caregiving. In this regard, the nurse aides were relatively less competent in terms of the logicality, comprehensibility, and overall analysis of a patient’s condition. Since nurse aides play a major front-line role in the caregiving workforce, Chen and Lin [12] suggested that nurse aides should be allowed to participate in the process of developing and revising patient care plans. Caregiving institutions should provide nurse aides with relevant training programs in order to enhance their effectiveness and task mastery, thus meeting the needs of caregiving institution residents [23].

According to the results, the nurses reported the nurse aides to be most competent in “work ethics”, “interpersonal relationships and communication”, and “caregiving competence”. In particular, the nurse aides reported themselves to be competent in the items “not doing anything that would harm patients”, “complete obligatory caregiving duties diligently”, and “respects the dignity and privacy of patients”. The nurses were highly affirmative about assigning routine and laborious tasks to the nurse aides. This finding is similar to that of a previous study [20]. On the other hand, the nurses felt that the nurse aides were least competent for the items “designing activities that meet the needs of patients”, “performing a simple evaluation of the effectiveness of implementing an activity”, and “communicating and cooperating with each department in the organization”. This finding is in line with that of Chen and Lin’s study [12], that is, nurse aides were perceived to be least competent in patient activity design. According to Liou [25], many nurses and nurse aides found it difficult to communicate or share patient-related information with each other or had adopted a reserved attitude. The study inferred that several factors contributed to this outcome, such as the lower level of professional knowledge of nurse aides, the qualities possessed by the nurse aides, and the lack of initiative and poor service attitude of nurse aides. In addition, the mentalities of the chiefs of institutional facilities, employee turnover, and poor workforce arrangements had also resulted in inadequate activity design competence and poor cross-departmental communication.

This study revealed that there are no significant differences between the self-reported and the nurses’ perceived overall work competence of nurse aides. The competence of the nurse aides in “activity design” as perceived by the registered nurses was lower than the nurse aides’ own perceptions. This finding is similar to that reported by Chen and Lin [12] in their study, that is, among all the prerequisite competencies of nurse aides, competence in activity design had the lowest score. This could be due to the higher expectations of the nurses toward the nurse aides, as according to the concept of holistic care, the nurses not only expect the nurse aides to complete basic tasks such as providing physical care and routine care and doing chores, but also expect them to be able to provide assistance in solving patient-related problems, assist in handling sudden events, understand the activity needs of residents, and guide patients to engage in simple activities. In Taiwan, nurse aide training programs often lack core courses pertaining to activity design. Moreover, the Taiwan “Long-Term Care Services Act” does not have any defined regulations on activity design in institutional long-term care services and practical settings. Furthermore, the lack of training among nurses and nurse aides regarding rehabilitation activity guidance or activity design has contributed to the gap in the nurse aides’ self-reported competence in activity design and that perceived by nurses.

Our results demonstrate that in general, the nurse aides and nurses had positive responses on the nurse aides’ work competence. Aued et al. and Boscart et al. [7,26] concurred that work experience is a primary component of a nurse aide’s clinical competence. The longer a nurse aide’s tenure at the same work setting, the more experience they have, and the better their clinical competence and overall quality of care. Up to two-thirds of the nurse aides in this study had at least six years of experiences, and those giving care to patient groups had been working in the same setting, which enabled them to easily communicate with the nurses, who were able to understand and comprehend the work status of the nurse aides. Consequently, both parties are able to increase their satisfaction with each other’s work competence. Nevertheless, based on the recommendations of Chen and Lin [12], professional physical therapists or occupational therapists should be the ones assessing the rehabilitation activites required by patients and designing customized activity plans. Nurse aides, on the other hand, could provide assistance in activity design and guide patients to engage in such rehabilitation activities. This approach would create a win-win scenario for all the healthcare providers who are involved.

### Limitations

Since this study was conducted at a hospital-based nurse institution in Taiwan and purposive sampling was employed, the results cannot be generalized to nurses and nurse aides in non-institutional settings and regional hospitals. The nurse aides of the hospital were outsourced workers who were jointly managed by the contractors and unit supervisors of the hospital. Thus, the responses are easily influenced by the nurse aides’ job performance. Moreover, the structural questionnaire design made it difficult for the participants to make specific responses or allow us gain an in-depth understanding of the scale items. We expect that more in-depth discussions should be made in the future in order to understand the relationships between the scale items.

## 5. Implication for Practice

The services provided by nurse aides are generally parallel to those of other healthcare providers, which decreases their interactions with cross-disciplinary teams. Since the caregiving tasks of nurse aides are mostly laborious and their trainings lack complete and systematic contents, they are easily neglected and excluded from cross-disciplinary teams. Nonetheless, nurse aides are a major part of the front-line caregiving workforce and are still members of institutional caregiving teams. In addition to providing relevant training, this study proposes that nurse aides should be incorporated into cross-disciplinary teams and be allowed to participate in resident care plans, as this enables them to acquire an extensive knowledge of suitable treatments. Additionally, allowing nurse aides to jointly formulate care plans can facilitates the development of their comprehensibility, overall analytical capabilities, and ability to provide appropriate care to residents.

Activity design should be handled by other healthcare providers, while nurse aides should be utilized based on their capabilities. Residents in nursing institutions or nursing homes mostly have to deal with cognitive impairments or impairments in activities of daily living, which reduces their willingness to engage in activities and thereby result in functional decline. The implementation of rehabilitation activities as an intervention can counter frailty and also prevent or delay the onset of disabilities. In other words, regular physical activities or proactive intervention measures are the best means of preventing functional decline in a resident. This study found that the nurse aides generally had a low education level, which may restrict their abilities to assess and design activities that are tailored to residents’ needs. Therefore, we suggest that activity design should be conducted by professionals such as senior social workers or physical therapists, while nurse aides should provide assistance by contributing to activity design and guiding residents to engage in simple activities. This would help the residents to perform rehabilitation exercises or engage in other relevant activities that meet their needs.

## 6. Conclusions

The results indicated that the nurse aides had room for improvement in terms of activity design and problem solving. We suggest that the nurse aides should be incorporated into institutional cross-disciplinary teams in practice. The lack of competence in activity design of the nurse aides as perceived by the nurses was significantly higher than the self-perceptions of the nurse aides. Owing to limitations in the nurse aides’ educational backgrounds and professional training, it is suggested that activity design should be handled by professionals such as senior social workers or physical therapists, while nurse aides should play the role of assistants.

## Figures and Tables

**Table 1:** Demographic variables of nurse aides (*N* = 72).

Variable	Item	*N*	%
Gender	Male	11	15.28
	Female	61	84.72
Nationality	Taiwanese	68	94.44
	Vietnamese	3	4.17
	Other	1	1.39
Religion	Buddhism	37	51.39
	Taoism	11	15.28
	Christianity	5	6.94
	Catholicism	1	1.39
	Other	18	25.00
Education level	Elementary school or below	21	29.17
	Senior high school or vocational high school	37	51.39
	College or above	14	19.44
Marital status	Married	49	68.06
	Widowed	7	9.72
	Single	14	19.44
	Other	2	2.78
Tenure	<1 year	3	4.17
	1–2 years	15	20.83
	3–4 years	5	6.94
	5–6 years	2	2.78
	6 years and above	47	65.28
Salary range	<NT$10,000	1	1.39
	NT$10,000–20,000	12	16.67
	NT$30,000–40,000	59	81.94

**Table 2:** Demographic variables of registered nurses (*N* = 102).

Variable	Item	*N*	%
Gender	Male	2	1.96
	Female	100	98.04
Education level	Senior high school or vocational high school	2	1.96
	College or above	100	98.04
Marital status	Married	36	35.29
	Single	63	61.77
	Other	3	2.94
Tenure	1–2 years	21	20.59
	3–4 years	14	13.73
	5–6 years	5	4.90
	6 years and above	62	60.78

**Table 3:** Work competence scale self-reported by nurse aides (*N* = 72).

Subscale	Mean	SD
1. Interpersonal relationships and communication	4.34	0.66
2. Problem solving	4.28	0.78
3. Caregiving competence	4.32	0.72
4. Work ethics	4.45	0.80
5. Personal and professional growth	4.50	0.66
6. Managerial capabilities	4.40	0.74
7. Activity design	4.30	0.83
Total	4.36	0.67

**Table 4:** Nurse aides’ work competence evaluated by registered nurses (*N* = 102).

Subscale	Mean	SD
1. Interpersonal relationships and communication	4.36	0.62
2. Problem solving	4.15	0.72
3. Caregiving competence	4.36	0.74
4. Work ethics	4.61	0.57
5. Personal and professional growth	4.32	0.68
6. Managerial capabilities	4.25	0.70
7. Activity design	3.89	0.84
Total	4.29	0.59

**Table 5:** Differences between registered nurses and nurse aides.

Subscale	Job Title	Mean	SD	*t*-Value	*p*
Overall work competence	RN	4.29	0.59	−0.69	0.49
	NA	4.36	0.67		
Interpersonal relationships and communication	RN	4.36	0.62	0.22	0.82
	NA	4.34	0.66		
Problem solving	RN	4.15	0.72	−1.17	0.24
	NA	4.28	0.78		
Caregiving competence	RN	4.36	0.74	0.39	0.70
	NA	4.32	0.72		
Work ethics	RN	4.61	0.57	1.47	0.14
	NA	4.45	0.80		
Personal and professional growth	RN	4.32	0.68	−1.82	0.07
	NA	4.50	0.66		
Managerial capability	RN	4.25	0.70	−1.33	0.19
	NA	4.40	0.74		
Activity design	RN	3.89	0.84	−3.20	0.002 **
	NA	4.30	0.83		

** *p* < 0.01. RN: Registered nurse. NA: Nurse aide.

## Data Availability

Data sharing is not applicable to this article as no new data were created or analyzed in this study.
